# Comparative acute toxicity of gallium(III), antimony(III), indium(III), cadmium(II), and copper(II) on freshwater swamp shrimp (*Macrobrachium nipponense*)

**DOI:** 10.1186/0717-6287-47-13

**Published:** 2014-04-01

**Authors:** Jen-Lee Yang

**Affiliations:** Department of Life Science, Chinese Culture University, Taipei 111, Taiwan

**Keywords:** *Macroburachium nipponense*, Cu, Cd, Sb, Ga, In, LC_50_

## Abstract

**Background:**

Acute toxicity testing were carried out the freshwater swamp shrimp, *Macrobrachium nipponense*, as the model animal for the semiconductor applied metals (gallium, antimony, indium, cadmium, and copper) to evaluate if the species is an suitable experimental animal of pollution in aquatic ecosystem.

**Results:**

The static renewal test method of acute lethal concentrations determination was used, and water temperature was maintained at 24.0 ± 0.5°C. Data of individual metal obtained from acute toxicity tests were determined using probit analysis method. The median lethal concentration (96-h LC_50_) of gallium, antimony, indium, cadmium, and copper for *M. nipponense* were estimated as 2.7742, 1.9626, 6.8938, 0.0539, and 0.0313 mg/L, respectively.

**Conclusions:**

Comparing the toxicity tolerance of *M. nipponense* with other species which exposed to these metals, it is obviously that the *M. nipponense* is more sensitive than that of various other aquatic animals.

## Background

Semiconductor manufacturing has become a leading industry in some developing countries. Gallium, antimony, indium, cadmium, and copper are essential transition metals that are widely used for the manufacture of integrated circuits, electroplating, and photoelectric appliances. These metals are released into the environment during manufacturing processes such as etching, wet polishing, and cleaning operations, which may produce much potentially hazardous waste [1, 2]. Accidental industrial spills may lead to high concentrations of metal compounds in water, which have both acute and chronic toxic effects on aquatic organisms. Because these heavy metals do not degrade and thus accumulate in ecosystems, their toxic effects may be found at the molecular, cellular, and histological levels, even impacting homeostasis in organisms [3, 4]. Individual components of heavy metals have been reported by different authors to have varying toxicological effects on aquatic organisms, and deaths of animals have also been reported at various concentrations [5]. Many studies have revealed that these metal compounds possess apoptotic, and carcinogenic properties [6, 7].

Freshwater animals can be used for controlling pollution in three ways, involving three different time frames: (1) the determination of water quality criteria from which standards can be established, (2) monitoring the health of populations of fish in the field or in a hatchery, (3) providing an early warning system for potential harm to the aquatic environment [8, 9]. Both acute and chronic toxicity tests provide detailed information for assessing environmental stress [10]. Acute toxicity results in particular provide practical critical values that can be used for establishing tentative water quality criteria related to novel toxicants. Suitable model species are needed to evaluate the water quality in aquatic ecosystems [11].

Freshwater swamp shrimp (*Macrobrachium nipponense*) is a common aquatic invertebrate widely distributed in the downstream of rivers throughout the eastern Asia-Pacific [12], and it is a dominant species in stream ecosystem near semiconductor manufacturing districts in Taiwan. The purpose of this study was to assess the acute lethal toxicity of concentrations of gallium, antimony, indium, cadmium, and copper on juvenile *M. nipponense* under laboratory conditions in order to determine specific safety concentrations.

## Results

No mortality was detected in the control groups during laboratory static renewal tests (96-h acute toxicity) of the five metals. We recorded mortality in whole exposure durations for freshwater swamp shrimp, *Macrobrachium nipponense*, exposed to varying concentrations of gallium, antimony, indium, cadmium, and copper. Results demonstrated a positive relationship between the mortality rates of the exposed *M. nipponense* and the concentrations of metals in the testing solutions. It is clear that the higher the concentration, the shorter the median lethal concentration (24-h, 48-h, and 96-h LC_50_) of the five metals to *M. nipponense*, as presented in Table 1. Based on 96-h LC_50_ values, the ranking of the five metals from most toxic to least toxic was: copper, cadmium, antimony, gallium, and indium.

**Table 1:** **Median lethal concentrations (LC**_**50**_ **) of gallium, antimony, indium, cadmium, and copper to** ***Macrobrachium nipponense***

	LC _50_(mg/L)		
	24 h	48 h	96 h
Galliumm (III)	12.6871	6.1262	2.7742
	(5.6189-28.6465)	(4.0147-9.3481)	(1.8210-4.2264)
antimony (III)	7.4570	3.7468	1.9626
	(2.9343-18.9501)	(2.5673-10.2216)	(1.3884-2.7743)
indium (III)	21.5464	14.8985	6.8938
	(7.2018-64.4628)	(9.2185-24.0784)	(3.6560-12.9992)
cadmium (II)	0.7109	0.1138	0.0539
	(4.8657-10.3881)	(0.0631-0.2053)	(0.0284-0.1021)
copper (II)	0.3831	0.0959	0.0313
	(0.1811-0.8105)	(0.0686-0.1343)	(0.0174-0.0562)

The 95% confidence limits are given in parentheses.

**Figure 1:**
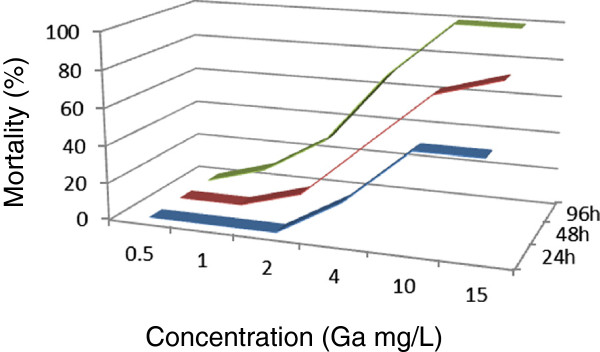
**Gallium lethality curves of** ***Macrobrachium nipponense*** **were determined at different gallium concentrations in the exposed environment.**

**Figure 2:**
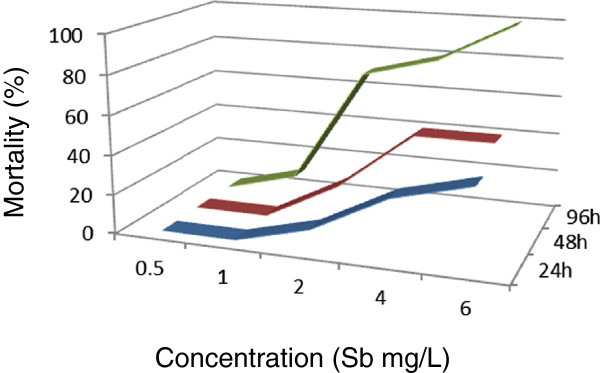
**Antimony lethality curves of** ***Macrobrachium nipponense*** **were determined at different antimony concentrations in the exposed environment.**

**Figure 3:**
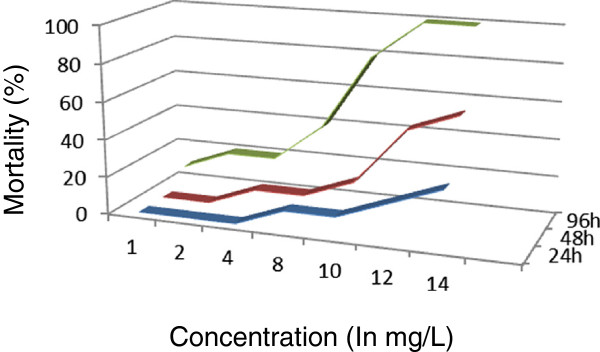
**Indium lethality curves of** ***Macrobrachium nipponense*** **were determined at different indium concentrations in the exposed environment.**

**Figure 4:**
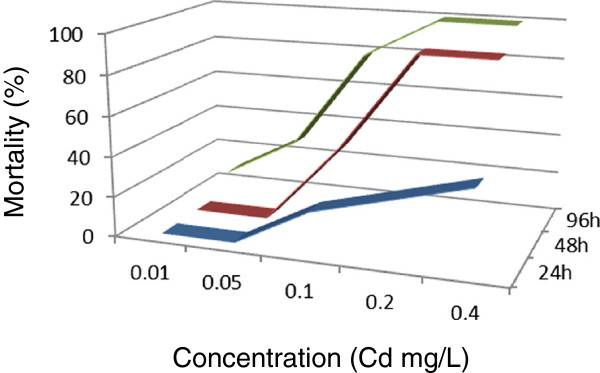
**Cadmium lethality curves of** ***Macrobrachium nipponense*** **were determined at different cadmium concentrations in the exposed environment.**

**Figure 5:**
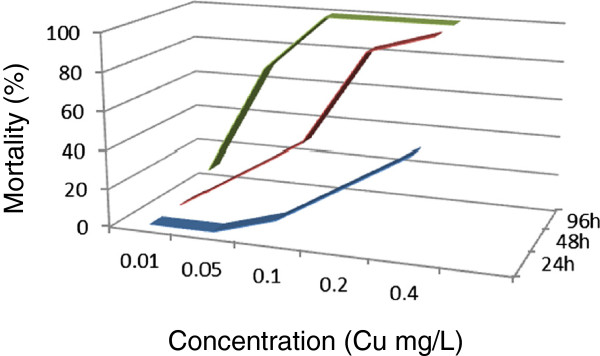
**Copper lethality curves of** ***Macrobrachium nipponense*** **were determined at different copper concentrations in the exposed environment.**

In toxicity testing of gallium, no mortality was observed in the group of *M. nipponense* exposed to 0.5 mg/L within 96 hours (Figure 1). The 96-h LC_50_ of the *M. nipponense* was determined to be 2.7742 mg/L, with upper and lower limits of 1.8210 and 4.2264 mg/L. Except for indium, the toxicity of gallium to *M. nipponense* was found to be less than that of the other metals in this study. One hundred percent mortality was detected in *M. nipponense* exposed to antimony in 6.0 mg/L after 96 hours (Figure 2). The 96-h LC_50_ value obtained for antimony was 1.9626 (1.3884-2.7743) mg/L. No mortality was recorded in *M. nipponense* exposed to indium in 4.0 mg/L solution in the first 24 hours (Figure 3). The 96-h LC_50_ value obtained was 6.8938 (3.6560 ~ 12.9992) mg/L for this metal. After 24 h, mortality was not observed for two of the metals (copper and cadmium) at concentrations of 0.05 mg/L (Figures 4 and 5). Compared to copper, a higher dose of cadmium is needed to obtain 48-h and 96-h LC_50._ Copper had stronger toxicity than cadmium in the present study.

## Discussion

Lin and Hwang [13] estimated the 96-h LC_50_ of gallium for tilapia larvae (*Oreochromis mossambicus*) to be 14.32 mg/L; this reveals that *M. nipponense* are more sensitive to gallium exposure than tilapia larvae. Betoulle [14] reported that gallium (III) accumulates in the head, kidney, and blood in juvenile common carp (*Cyprinus carpio*). Gallium also acts as a hepatotoxin and causes renal damage in treated *C. carpio* [15, 16].

Lin and Hwang [17] reported the 96-h LC_50_ value of antimony for tilapia larvae (*Oreochromis mossambicus*) as 18.9 mg/L, and observed retardation in body growth at sublethal concentrations. The present study found *M. nipponense* to be more susceptible than tilapia larvae to acute antimony toxicity. With the exception of deleterious information on the effect on *Daphnia magna*, little or no research is available documenting the effect of antimony on freshwater invertebrates [18].

Lin and Hwang [19] reported the 96-h LC_50_ value of indium for tilapia larvae (*Oreochromis mossambicus*) as 19.5 mg/L, demonstrating that tilapia larvae are more able to tolerate to indium exposure than *M. nipponense*. The indium value of 6.8938 mg/L was higher than that of gallium and antimony for the same stage of *M. nipponense*. This study is the first to examine the effects of indium on aquatic invertebrates.

Both copper and cadmium are highly toxic for aquatic organisms; effects of short- and long-term exposure to these two metals have already been reported in a number of studies [20–22]. Wu and Chen [23] showed the 96-h LC_50_ value of cadmium for white shrimp (*Litopenaeus vannamei*) is 1.07 mg/L; Tan and Wang [24] also promoted a biotic model for predicting the acute toxicity of cadmium to daphnids under different calcium and pH conditions. As for copper, Karan et al. [25] reported significant changes in metabolic enzymes in gills, livers, and blood of *C. carpio* exposed to sublethal concentrations of copper (96-h LC_50_ value: 0.64 mg/L). Acute lethal effects of copper were attributed to excess mucous covering the gill tissues, leading to the breakdown of respiratory function [26].

## Conclusions

Regulation of different activities involving heavy metals does exist in an industrial country like Taiwan [27]. However, scanning the existing data base for surveillance studies in high risk populations, which mainly involve aquatic fauna and flora, turned up nothing. The natural habitats of *M. nipponense* are frequently polluted by industrial wastewater. To maintain the balance of the local river ecosystems it is essential to know the susceptibility to these pollutants. We conclude that *M. nipponense* seem to be a promising candidate for evaluating freshwater quality, as they are sensitive to these metals. Moreover, no acute lethal effects were seen at concentrations of 0.28 mg/L (gallium), 0.20 mg/L (antimony), 0.69 mg/L (indium), 0.005 mg/L (cadmium), and 0.003 mg/L (copper). These findings are in good agreement with the concept of a safe level (one-tenth of the 96-h LC_50_ value) as described by Sprague [28], and we propose them as biologically safe concentrations which can be used for establishing tentative water quality criteria with *M. nipponense* of the same size.

## Methods

Freshwater swamp shrimp (*Macrobrachium nipponense*) were obtained from the local commercial suppliers. Shrimp were transported to a glass aquarium in our laboratory which was equipped with a water-cycling device; dechlorinated tap water (with a pH of 7.4 ~ 8.1, dissolved oxygen (DO) of 7.0 ~ 7.7 mg/L, and hardness of 38 ~ 45 mg CaCO_3_ /L) was used during the entire experiment. The temperature was maintained at 24.0 ± 0.5°C, with a 12-h light and 12-h dark photoperiod. Shrimp were acclimated for two weeks and fed an aquarium shrimp mixture every day. Shrimp (0.42 ± 0.17 cm in fork length) were used for acute toxicity tests in the initial experiments. Gallium sulfate (III) and antimony chloride (III) were purchased from Alfa Aesar (Ward Hill, MA, USA). Indium chloride (III), copper sulfate (II), and cadmium chloride (II) were purchased from Sigma (St. Louis, MO, USA). All metal compounds had a purity of 99% or greater. Stock solutions were prepared in deionized water (1000 mg/L test chemical in 0.1% nitric acid).

Laboratory static renewal tests were conducted to determine the median lethal concentration (LC_50_) for *M. nipponense*. Ten animals of similar size were randomly sampled and placed in 10-L glass beakers. After 24 h of acclimatization, the *M. nipponense* were exposed to different concentrations of gallium (0, 0.3, 0.5, 1.0, 2.0, 4.0, 6.0, 8.0, and 10.0 mg/L), antimony (0, 0.5, 1.0, 2.0, 4.0, 8.0, 12.0, 14.0, and 16.0 mg/L), indium (0, 0.5, 1.0, 2.0, 4.0, 8.0, 12.0, 14.0, and 16.0 mg/L), cadmium (0, 0.002, 0.005, 0.01, 0.05, 0.1, 0.2, 0.4, and 0.6 mg/L), and copper (0, 0.002, 0.005, 0.01, 0.05, 0.1, 0.2, 0.4, and 0.6 mg/L) for 96 h or more. The control and each treated group were run in duplicate. During the experiment, dead animals were removed, with mortality recorded after 24, 48, and 96 h. The LC_50_ of every test chemical with 95% confidence limits were calculated for *M. nipponense* using a Basic program from the probit analysis described by Finney [29].

## References

[1] Robinson AL (1983). GaAs readied for high-speed microcircuits. Science.

[2] Sturgill JA, Swartzbaugh JT, Randall PM (2000). Pollution prevention in the semiconductor industry through recovery and recycling of gallium and arsenic from GaAs polishing wastes. Clean Prod Proc.

[3] Buikema Al JR, Niederlehner BR, Cairns JJR (1982). Biological monitoring. Part IV Toxicity testing. Water Res.

[4] Gopalakrishnan S, Thilagam H, Raja PV (2007). Toxicity of heavy metals on embryogenesis and larvae of the marine sedentary Polychaete Hydroides elegans. Arch Environ Contam Toxicol.

[5] Woltering DM (1984). The growth response in fish chronic and early life stage toxicity tests: A critical review. Aquat Toxicol.

[6] Bustamante J, Lennart D, Marie V, Bruce F, Sten O (1997). The semiconductor elements arsenic and indium induce apoptosis in rat thymocytes. Toxicology.

[7] Marisa DP, Parrish AR (2003). Metal-induced apoptosis: mechanisms. Mechan Muta.

[8] Heath AG (1987). Water pollution and fish physiology.

[9] Paez-Osuna F, Tron-Mayen L (1996). Concentration and distribution of heavy metals in tissues of wild and farmed shrimp *Penaeus vannamei* from the northwest coast of Mexico. Environ Internl.

[10] Mehrle PM, Mayer FL (1980). Clinical tests in aquatic toxicology: state of the art. Environ Health Perspect.

[11] Stephenson RR (1982). Aquatic toxicology of cypermethrin. I. Acute toxicity to some freshwater fish and invertebrates in laboratory tests. Aquatic Toxicol.

[12] Yang P, Zhang H, Chen LQ, Ye JY, Yu N, Gu ZM, Song DX (2007). Genetic structure of the oriental river prawn (*Macrobrachium nipponense*) from the Yangtze and Lancang rivers, inferred from COI gene sequence. Zool Res.

[13] Lin HC, Hwang PP (1998). Acute and chronic effects of gallium chloride (GaCl_3_) on tilapia (*Oreochromis mossambicus*) larvae. Bull Environ Contam Toxicol.

[14] Betoulle (2002). In vivo and in vitro modulation of carp (*Cyprinus carpio*) phagocyte oxidative burst activity by gallium. J Toxicol Environ Health.

[15] Yang JL, Chen HC (2003). Serum enzyme activities and hepatocyte ultrastructure of common carp after gallium exposure. Zool Stud.

[16] Yang JL, Chen HC (2003). Growth retardation and histopathology of common carp (*Cyprinus carpio*) exposed to gallium. Bull Environ Contam Toxicol.

[17] Lin HC, Hwang PP (1998). Acute and chronic effects of antimony chloride (SbCl_3_) on tilapia (*Oreochromis mossambicus*) larvae. Bull Environ Contam Toxicol.

[18] Filella M, Williams CPA, Belzile N (2009). Antimony in the environment: knowns and unknowns. Environ Chem.

[19] Lin HC, Hwang PP (1998). Acute and chronic effects of indium chloride (InCl_3_) on tilapia (*Oreochromis mossambicus*) larvae. Bull Environ Contam Toxicol.

[20] Qu RJ, Wang XH, Feng MB, Li Y, Liu HX, Wang LS, Wang ZY (2013). The toxicity of cadmium to three aquatic organisms (*Photobacterium phosphoreum*, *Daphnia magna* and *Carassius auratus*) under different pH levels. Ecotoxic Environ Safe.

[21] Wong CKC, Wong MH (2000). Morphological and biochemical changes in the gills of tilapia (*Oreochromis mossambicus*) to ambient cadmium exposure. Aquat Toxicol.

[22] Yang HN, Chen HC (1996). The influence of temperature on the acute toxicity and sublethal effects of copper, cadmium and zinc to Japanese eel, *Anguilla japonica*. Acta Zool Taiwanica.

[23] Wu JP, Chen HC (2004). Effects of cadmium and zinc on oxygen consumption, ammonium excretion, and osmoregulation of white shrimp (*Litopenaeus vannamei*). Chemosphere.

[24] Tan QG, Wang WX (2013). Acute toxicity of cadmium in *Daphnia magna* under different calcium and pH conditions: importance of influx rate. Environ Sci Technol.

[25] Karan V, Vitorvic S, Tutundzic V, Poleksic V (1998). Functional enzymes activity and gill histology of carp after copper sulfate exposure and recovery. Ecotoxicol Environ Saf.

[26] Vutukuru SS, Suma CH, Madhavi KR, Juveria PJS, Rao JV, Anjaneyulu Y (2005). Studies on the development of potential biomarkers for rapid assessment of copper toxicity to freshwater fish using esomus danricus as Model. Int J Environ Res Public Health.

[27] Chen WH (2006). Gallium, indium, and arsenic pollution of groundwater from a semiconductor manufacturing area of Taiwan. Bull Environ Contamin Toxicol.

[28] Sprague JB (1971). Measurement of pollutant toxicity to fish. III. Sublethal effects and “safe” concentrations. Water Res.

[29] Finney DJ (1971). Probit Analysis.

